# Changes in High-Risk HPV Infection Prevalence and Associated Factors in Selected Rural Areas of China: A Multicenter Population-Based Study

**DOI:** 10.3389/fmed.2022.911367

**Published:** 2022-07-12

**Authors:** Yan-Qin Yu, Ming-Yue Jiang, Le Dang, Rui-Mei Feng, Mohamed S. Bangura, Wen Chen, You-Lin Qiao

**Affiliations:** ^1^Department of Cancer Epidemiology, National Cancer Center/National Clinical Research Center for Cancer/Cancer Hospital, Chinese Academy of Medical Sciences and Peking Union Medical College, Beijing, China; ^2^Department of Public Health and Preventive Medicine, Baotou Medical College, Baotou, China; ^3^School of Public Health, Fujian Medical University, Fuzhou, China; ^4^School of Public Health, Dalian Medical University, Dalian, China; ^5^Chinese Academy of Medical Sciences and Peking Union Medical College School of Population Medicine and Public Health, Beijing, China

**Keywords:** high-risk human papillomavirus, cervical cancer, genotype, risk factors, China demonstration rural areas

## Abstract

**Background:**

The Chinese government has taken action to prevent cervical cancer by implementing the National Cervical Cancer Screening Programme in Rural Areas (NACCSPRA), which was launched in 2009. Numerous studies have demonstrated that long-term cervical cancer screening alters human papillomavirus (HPV) infection rates and cervical disease detection. Nearly 80 million women have been screened over 10 years, representing <30% of the target population; however, in some rural areas, such as Ordos City of Inner Mongolia Autonomous Region, Xiangyuan County of Shanxi Province, and Jinyun County, and Jingning County of Zhejiang Province, programs for prevention and treatment of cervical cancer have been implemented. Numerous studies have demonstrated that long-term cervical cancer screening alters rates of human papillomavirus (HPV) infection and cervical disease detection. In this study, we aimed to determine the infection rates of high-risk HPV (hrHPV) and the detection rate of cervical lesions; and changes in factors associated with cervical cancer, to provide scientific data to inform efforts to eliminate cervical cancer in rural areas.

**Methods:**

This was a cross-sectional, population-based, and multi-center survey. Populations from three rural areas of China (Ordos City of Inner Mongolia Autonomous Region, Xiangyuan County of Shanxi Province, and Jinyun County and Jingning County of Zhejiang Province) were selected and 9,332 women aged 20–64 years old were invited to participate in cervical cancer screening by both cytology and HPV testing. The outcomes assessed were: infection rates with hrHPV, HPV16, 18, 16/18, and other 12 hrHPV types (HPV 31,33,35,39,45,51,52,56,58,59,66 and 68); detection rates of cytological and histological lesions; and factors associated with HPV infection.

**Results:**

A total of 9,217 women aged 45.62 ± 8.02 years were included in this study. Infection rates with hrHPV, HPV 16, 18, 16/18, and other 12 hrHPV types were 16.3%, 3.0%, 1.5%, 4.3%, and 13.6%, respectively. There were significant differences among the age-specific HPV infection rates (*P* < 0.05). Infection rates with hrHPV, 16, 18, 16/18, and the other 12 hrHPV types showed a single peak infection mode, with a peak age of 56–65 years old. Age, marital status, number of live births, education level, reproductive disease history, and a history of alcohol consumption were risk factors for hrHPV infection. The detection rate of cytological abnormalities was 12.98% in the study and was higher in women older than 56 years old. The detection rates of cervical intraepithelial neoplasia CIN2+ and CIN3+ in the population were 1.45% and 0.77%, respectively. The highest incidence rates of CIN2+ and CIN3+ were 32.12% and 17.51%, respectively, in the 41–45 years old group.

**Conclusion:**

Infection rates with hrHPV, HPV16, and cervical lesions among our screening population were lower than the mean level in rural areas of China. Infection rates with hrHPV, HPV16, 18, and 16/18 showed a single-peak infection pattern, with the peak age of infection being 56-65 years old. Risk factors for hrHPV infection were age, history of alcohol consumption, marital status, reproductive diseases, education level, and the number of live births. Based on these data, we recommend that cervical cancer screening be offered to women older than 30 years in rural areas, particularly those aged 41–45 years.

## Introduction

Cervical cancer is ranked third among gynecological malignancies in terms of both estimated new cases and deaths of women worldwide. An estimated 604,000 new cervical cancer cases and 342,000 deaths were reported globally in 2020 (1). In China, there were up to 110,000 and 60,000 of new cases and deaths from cervical cancer, respectively, in 2020 (2), representing increases of 3.5% and 23.0% relative to 2018 (3). Hence, prevention and treatment of cervical cancer are urgent, particularly in China.

There has been heavy investment in cervical cancer prevention and control in China in recent years; however, the goal of eliminating cervical cancer, especially in rural areas, remains some way from being achieved (4). The Chinese government has taken action to prevent cervical cancer by implementing the National Cervical Cancer Screening Program in Rural Areas (NACCSPRA), which was launched in 2009 to provide free annual screening for 10 million women aged 35–64 in rural China (5, 6). Over the past decade, screening areas and population coverage have been expanding, with a screening rate for rural women from 2016 to 2018 of 26% (7), which remains far from the 70% screening target proposed by the World Health Organization (WHO). Due to imbalances in economic development, health levels, HPV infection rates, risk factors, and disease detection rates in rural areas of China, successful implementation of the cervical cancer elimination plan for China is challenging in these areas, particularly the prevention and treatment of cervical cancer. The detection and screening rates for precancerous cervical lesions were raised in rural China (8). Good systems for prevention and treatment of cervical cancer have been achieved in some rural areas of China, such as Ordos City, which has high rates of cervical cancer incidence. As the first city in China to implement a policies of screening for cervical cancer in all women aged 35–64 years and to conduct HPV vaccine immunization for all girls aged 13–18 years, the WHO considered the Ordos of the city in China likely to be first eliminate cervical cancer (9)^1^, and the region has a high population of people with Mongol ethnicity, who have a higher incidence of cervical cancer. Further, due to its implementation of cervical cancer screening for almost 30 years, Xiangyuan County in Shanxi Province is a rural area that demonstrates the potential for the prevention and treatment of cervical cancer in China, and this rural area was also a clinical experimental site for the bivalent, quadrivalent, and 9-valent HPV vaccines (10). Jinyun County and Jingning County in Zhejiang Province also have a good record of cervical cancer prevention and treatment. In addition, it was the clinical experimental site for the HPV screening kit.

HPV infection rates, particularly the HPV 16 infection rate, were altered by the implementation of cervical cancer screening measures after ten years follow-up (11). The prevention and treatment experience of cervical cancer in Australia and other countries have confirmed that cervical cancer screening and HPV vaccination reduce the detection rate of cervical lesions. Compared with similar published studies, this study focused on the change of selected rural areas, where successful implementation of a cervical cancer elimination plan was introduced and had higher cervical cancer incidence and mortality rates in rural areas of China. There were unreported changes in HPV infection and cervical lesion detection rates and factors influencing these selected rural areas, especially after HPV vaccination in the market. Therefore, we conducted this study to explore the extent of prevention and treatment of cervical cancer in rural China by comparing how HPV infection rates, cervical lesions detection rates, and factors influencing HPV infection have changed. Furthermore, we investigated the effectiveness and relationship with age of cervical cancer screening for women in areas with high incidence rates of cervical cancer to provide a basis for design of follow-up cervical cancer screening strategies. The primary purpose of this study was to evaluate changes in HPV infection and cervical lesion detection rates and factors influencing these in screened women aged 20–65 years in rural areas of China with high incidence of cervical cancer: Ordos City of Inner Mongolia Autonomous Region, Xiangyuan County of Shanxi Province, and Jinyun County, and Jingning County of Zhejiang Province.

## Methods

### Setting

This was a multicenter, population-based, and cross-sectional study conducted in rural areas of China from 2016 to 2019. Three rural areas were chosen based on their high incidence rates of cervical cancer, including the Ordos, Inner Mongolia (Hang jin banner and Yi jinholo banner), Shanxi Province (Xiangyuan Country), and Zhejiang Province (Jinyun County and Jingning County). A total of three tertiary hospitals and five maternal and child health hospitals were selected. Ethics approval was obtained from the Cancer Hospital, Chinese Academy of Medical Sciences (No. 16-013/1092), and the study was approved by all institutional review boards of the participating hospitals.

### Study Population

**Figure 1:**
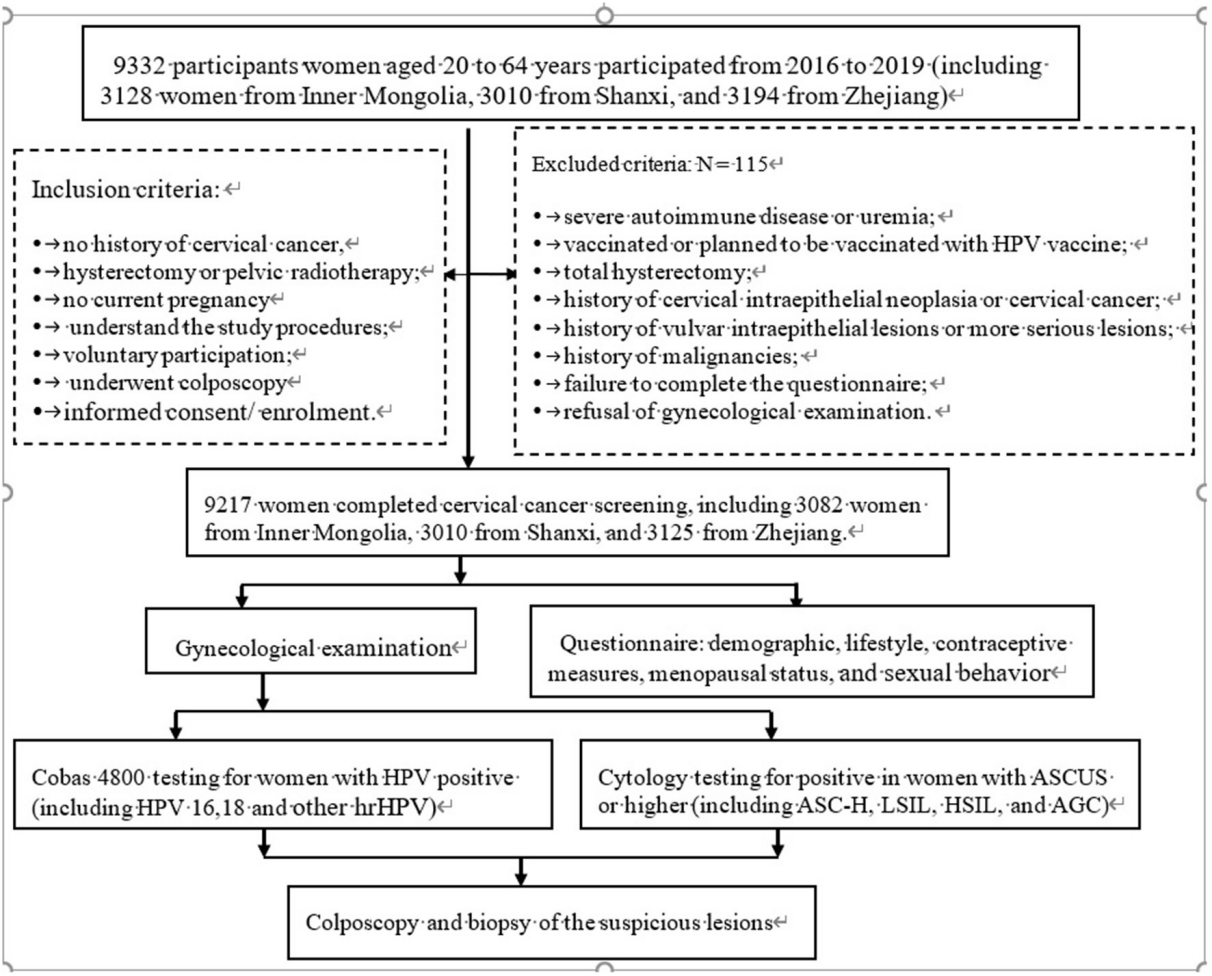
The flow chart of the study procedures carried out on the sites. ASCUS, atypical squamous cells of undetermined significance; ASC-H, higher including low-grade squamous cell-cannot exclude high-grade squamous intraepithelial lesion; LSIL, low-grade intraepithelial lesion; HSIL, high-grade squamous intraepithelial lesion; AGC, atypical glandular cell; NILM, negative for intraepithelial lesion or malignancy.

In the initial stage of the study, 9,332 women participated, while 9,217 eligible women aged 20 to 64 years who lived in villages or sub-districts participated in the questionnaire survey, gynecological examination, and laboratory testing. All eligible women provided informed consent before enrolment. The investigators carried out procedures, inspecting whether the women complete the questionnaires or met the inclusion and exclusion criteria (Figure 1).

### Procedure

The survey was conducted face-to-face by trained interviewers. If patients had difficulty reading and completing the scales, trained interviewers helped them read and explain, or family members helped them answer questions. Information collected included: demographic (birth date, sex, location, occupational situation, marital status, education, and annual household income); and other factors, including a history of disease, pregnancy, reproduction, lifestyle (smoking and alcohol consumption), contraceptive measures, menopausal status, and sexual behavior. A strict quality control scheme was adhered to throughout the entire investigation process, including data collection, filing, entry, and checking, revision, and data security. The trained interviewers checked the questionnaires immediately on completion to avoid missing items and logical errors. If the questionnaires had missing items or obvious logical mistakes (such as missing items and errors), the trained interviewers called the patient to amend them and check the information. All procedures were performed by trained local physicians, while the materials for and results of cytology and hrHPV analyses were provided by central hospitals.

### Cervical Cancer Screening Process

All women were tested by cytology and for hrHPV using Thin Prep medium and the Cobas 4,800 test (Roche Diagnostics). Women positive for either HPV 16/18 or other HPV genotypes with positive cytology results were deemed to have screened positive; colposcopy and biopsy of suspicious lesions were performed if necessary. Women positive for other HPV genotypes and negative on cytology, and those negative for HPV genotypes, were deemed to have screened negative. The results of cytology show atypical squamous cells of undetermined significance (ASCUS) or higher (including low-grade squamous cell-cannot exclude high-grade squamous intraepithelial lesion (ASC-H), low-grade intraepithelial lesion (LSIL), high-grade squamous intraepithelial lesion (HSIL), and atypical glandular cell (AGC), among others) indicated the need to undergo colposcopy and biopsy of the suspicious lesions. Women negative for intraepithelial lesion or malignancy (NILM) were screened negative, with follow-up observation to be carried out in 3 years.

According to CIN terminology, CIN was diagnosed as one of four stages: NILM, CIN grade 1 (CIN1), CIN2, CIN3; and cervical cancer as micro-invasive carcinoma, invasive carcinoma, and others.

Precancerous lesions diagnosed by cytology were classified into four stages: NILM, ASCUS, LSIL, and HSIL+.

### Statistical Analysis

A database was established using Microsoft Access 2007 software. Statistical analyses were performed in SPSS, version 28.0. A χ^2^ test was performed to compare proportions in subjects with specific characteristics and incidence rates of hrHPV, HPV16, 18, 16/18, and another 12 high-risk HPV types (31, 33, 35, 39, 45, 51, 52, 56, 58, 59, 66 and 68) and cytology. Linear trend tests were used to compare infection rates with total hrHPV, HPV16, 18, 16/18, and the 12 other hrHPV genotypes; detection rates of abnormal cells; and detection rates of cervical precancerous lesions, according to age group.

## Results

### Sociodemographic Information

Initial screening for cervical cancer was conducted in 9,332 women from rural areas, of which 115 were excluded for various reasons, including an unwillingness to consent, disapproval of gynecological examination, previous uterine surgery, and incomplete data. Finally, 9,217 women completed cervical cancer screening, including 3,082 women from Inner Mongolia, 3,010 from Shanxi, and 3,125 from Zhejiang. The mean age of the 9,217 women was 45.15 ± 8.74 years, and women aged 41–45-years-old accounted for 20.0% of the screened population. The largest age groups from Inner Mongolia, Shanxi Province, and Zhejiang Province in the screened populations were those aged 51–55 years-old (20.1%), 41–45 years old (19.5%), and 56–60 years old (9.4%), respectively. There were significant differences in age distribution, marital status, and educational level (*P* < 0.001) (Table 1).

**Table 1:** Basic characteristics of the study population.

**Characteristic**	**Total population, N (%)**	**Inner Mongolia, *N* (%)**	**Shanxi, *N* (%)**	**Zhejiang, N (%)**	***χ^2^***	***P*-value**
**Age (years)**
21–25	45 (0.5)	18 (0.6)	16 (0.5)	11 (0.4)	475.3	0.00
26–30	482 (5.2)	127 (4.2)	248 (8.0)	107 (3.4)		
31–35	922 (10.0)	190 (6.3)	488 (15.8)	244 (7.8)		
36–40	1,327 (14.4)	338 (11.2)	508 (16.5)	481 (15.4)		
41–45	1,844 (20.0)	576 (19.1)	601 (19.5)	667 (21.3)		
46–50	1,860 (20.2)	597 (19.8)	554 (18.0)	709 (22.7)		
51–55	1,570 (17.0)	606 (20.1)	405 (13.1)	559 (17.9)		
56–60	862 (9.4)	397 (13.2)	208 (6.7)	257 (29.8)		
61–65	305 (3.3)	163 (5.4)	52 (1.7)	90 (2.9)		
**Marital status**
Unmarried	10 (0.1)	0 (0.0)	3 (0.0)	7 (0.1)	90.6	0.00
Married	9,034 (98.0)	3,023 (98.1)	2,925 (97.2)	3,085 (98.7)		
Widowed	126 (1.4)	31 (1.0)	80 (2.7)	15 (0.5)		
Separated	12 (0.1)	2 (0.1)	2 (0.1)	8 (0.3)		
Divorced	32 (0.3)	19 (0.6)	3 (0.1)	10 (0.3)		
Other	4 (0.0)	4 (0.0)	0 (0.0)	0 (0.0)		
**Education Level**
Uneducated	641 (7.0)	483 (15.7)	55 (1.8)	103 (3.3)	1692.2	0.00
Primary school	1,885 (20.5)	599 (19.4)	827 (27.5)	459 (14.7)		
Junior school	3,706 (40.2)	689 (22.4)	1,662 (55.2)	1,355 (43.4)		
High school	1,398 (15.2)	432 (14.0)	373 (12.4)	593 (19.0)		
Undergraduate and above	1,587 (17.2)	879 (28.5)	93 (3.1)	615 (19.7)		

### Distribution of HPV Status

Among the women screened for cervical cancer, the positive rates for the hrHPV types, 16, 18, 16/18, and others (12 high-risk types) were 16.3%, 3.0%, 1.5%, 4.3%, and 13.6%, respectively. Further, the infection rates of HPV16, 18, 16/18, and other types in the total population differed significantly among age groups (*P* < 0.05), with the peak age of infection at 56–65 years old (Table 2). The infection rate of hrHPV (17.1% vs. 17.1%), HPV 16/18 (5.0% vs. 4.9%) and other 12 high-risk types (14.1% vs. 21.7%) among the screening population in Inner Mongolia and Shanxi Province were higher than those in Zhejiang Province (14.8%), HPV16 (1.6%), HPV16/18 (3.1%) and other 12 high-risk types (12.6%) (*P* < 0.05).There was no significant difference in infection rate of HPV 18 among the selected areas. Comparing the age groups, the infection rates of hrHPV and HPV 16/18 were statistically different among age groups in Inner Mongolia Autonomous Region and Shanxi Province (*P* < 0.05), except for the infection rates of HPV16. While infection rates of HPV16, 18, 16/18, and other types in the Zhejiang Province were statistically significant (*P* < 0.05), with the peak age of infection at 61–65 years old.

**Table 2:** High-risk HPV infection rate and genotype distribution by age group and areas, *N* (%).

	**Types**	**Amount**	**21–25**	**26–30**	**31–35**	**36–40**	**41–45**	**46–50**	**51–55**	**56–60**	**61–65**	***P*-value**
			**years**	**years**	**years**	**years**	**years**	**years**	**years**	**years**	**years**	
Total	HPV	1,504 (16.3)	5 (11.1)	68 (14.1)	137 (14.9)	195 (14.7)	232 (12.6)	283 (15.2)	292 (18.6)	216 (25.1)	76 (24.9)	0.00
	HPV16	278 (3.0)	0 (0.0)	11 (2.3)	28 (3.0)	33 (2.5)	53 (2.9)	45 (2.4)	46 (2.9)	43 (5.0)	19 (6.2)	0.00
	HPV18	136 (1.5)	1 (2.2)	7 (1.5)	12 (1.3)	16 (1.2)	19 (1.0)	20 (1.1)	34 (2.2)	20 (2.3)	7 (2.3)	0.00
	HPV16/18	400 (4.3)	1 (2.2)	18 (3.7)	36 (3.9)	48 (3.6)	71 (3.9)	65 (3.5)	75 (4.8)	61 (7.1)	25 (8.2)	0.00
	Other hrHPV	1,253 (13.6)	5 (11.1)	57 (11.8)	117 (12.7)	164 (12.4)	183 (9.9)	234 (12.6)	252 (16.1)	180 (20.9)	61 (20.0)	0.00
Inner mongolia	HPV	526 (17.1)	2 (11.1)	39 (15.7)	87 (17.8)	90 (17.7)	86 (14.3)	78 (14.1)	79 (19.5)	53 (25.5)	12 (23.1)	0.00
	HPV16	112 (3.6)	0 (0.0)	6 (2.4)	20 (4.1)	16 (3.1)	20 (3.3)	14 (2.5)	20 (4.9)	11 (5.3)	5 (9.6)	0.10
	HPV18	51 (1.7)	1 (5.6)	5 (2.0)	9 (1.8)	5 (1.0)	7 (1.2)	5 (0.9)	10 (2.5)	8 (3.8)	1 (1.9)	0.08
	HPV16/18	155 (5.0)	1 (5.6)	11 (4.4)	25 (5.1)	21 (4.1)	27 (4.5)	19 (3.4)	28 (6.9)	17 (8.2)	5 (11.5)	0.03
	Other hrHPV	434 (14.1)	2 (11.1)	33 (13.3)	45 (10.8)	75 (15.4)	67 (11.1)	64 (11.6)	62 (15.3)	44 (21.2)	10 (19.2)	0.01
Shanxi	HPV	516 (17.1)	2 (12.5)	13 (10.2)	23 (12.1)	46 (13.6)	71 (12.3)	101 (16.9)	110 (18.2)	109 (27.5)	41 (25.2)	0.00
	HPV16	101 (3.4)	0 (0.0)	4 (3.1)	4 (2.1)	10 (3.0)	20 (3.5)	17 (2.8)	13 (2.1)	26 (6.5)	7 (4.3)	0.02
	HPV18	48 (1.6)	0 (0.0)	1 (0.8)	1 (0.5)	5 (1.5)	5 (0.9)	5 (0.9)	8 (1.3)	14 (2.3)	10 (2.5)	0.32
	HPV16/18	147 (4.9)	0 (0)	5 (3.9)	5 (2.6)	15 (4.4)	25 (4.3)	25 (4.2)	26 (4.3)	36 (9.1)	10 (6.1)	0.00
	Other HPV	425 (14.1)	2 (12.5)	10 (7.9)	20 (10.5)	38 (11.2)	55 (9.5)	82 (13.7)	97 (16.0)	86 (21.7)	35 (21.5)	0.00
Zhejiang	HPV	462 (14.8)	1 (9.1)	16 (15.0)	27 (11.1)	59 (12.3)	75 (11.2)	104 (14.7)	103 (18.4)	54 (21.0)	23 (25.6)	0.00
	HPV16	65 (2.1)	0 (0.0)	1 (0.9)	4 (1.6)	7 (1.5)	13 (1.9)	14 (2.0)	13 (2.3)	6 (2.3)	7 (7.8)	0.03
	HPV18	37 (1.2)	0 (0.0)	1 (0.9)	2 (0.8)	6 (1.2)	7 (1.0)	7 (1.0)	10 (1.8)	2 (0.8)	2 (2.2)	0.00
	HPV16/18	98 (3.1)	0 (0.0)	2 (1.9)	6 (2.5)	12 (2.5)	19 (2.8)	21 (3.0)	21 (3.8)	8 (3.1)	9 (10.0)	0.03
	Other hrHPV	394 (12.6)	1 (9.1)	14 (13.1)	22 (9.0)	49 (10.2)	61 (9.1)	88 (12.4)	93 (16.6)	50 (19.5)	16 (17.8)	0.00

*HPV, including HPV 16, 18, Other HPV types (31,33,35,39,45,51,52,56,58,59,66 and 68) infection*.

### Risk Factors Associated With HrHPV Infection

Among the women screened for cervical cancer, HrHPV infection rates did not differ significantly according to disease history, age at menarche, menopause status, alcohol consumption and smoking (*P* > 0.05). However, the hrHPV infection rate differed significantly according to age at first pregnancy, age at first delivery, number of births and live births, method of contraception, menopause status, and the number of sexual partners (*P* < 0.05). Infection rates were higher in women who were younger at first pregnancy and delivery, had given birth 3–4 times, had 3–4 live births, had disease history, had ≥ 2 sexual partners, and used other method of contraception (including external ejaculation, vaginal medication, and fallopian tube blockage) (Table 3).

**Table 3:** Analysis of factors associated with high-risk HPV infections.

**Factor**		**Amount**	**HPV infection, *N* (%)**	**OR (95% CI)**
Education level	Uneducated	641	122 (19.03)	1
	Primary school	1,885	318 (16.87)	0.86 (0.68, 1.09)
	Junior school	3,706	583 (15.73)	0.79 (0.64, 0.99)
	High school	1,398	232 (16.59)	0.85 (0.66, 1.08)
	Undergraduate and above	1,587	249 (15.69)	0.79 (0.62, 1.01)
Age at menarche (years)	10–13	1,814	267 (14.71)	1
	14–17	6,600	1,100 (16.67)	1.15 (0.99, 1.33)
	≥ 18	744	135 (18.14)	1.28 (1.02, 1.61)
	Unclear	9	2 (22.22)	1.66 (0.34, 8.01)
Age at first pregnancy (years)	≤18	230	45 (19.56)	1
	19–21	2,978	536 (18.00)	0.90 (0.64, 1.27)
	22–24	3,542	553 (15.61)	0.76 (0.54, 1.07)
	25–27	1,806	273 (15.11)	0.73 (0.52, 1.04)
	≥ 28	574	79 (13.76)	0.66 (0.44, 0.98)
	No pregnancy	79	17 (21.51)	1.13 (0.60, 1.78)
	Refuse to answer	8	1 (12.50)	0.59 (0.07, 4.89)
Age at first delivery (years)	No delivery	106	24 (22.64)	1.11 (0.69, 1.80)
	≤20	1,052	219 (20.82)	1
	21–25	5,609	918 (16.37)	0.74 (0.63, 0.88)
	26–30	2,244	314 (13.99)	0.62 (0.51, 0.75)
	≥ 31	198	28 (14.14)	0.63 (0.41, 0.96)
	Refused to answer	8	1 (12.5)	0.54 (0.07, 4.44)
Number of pregnancies	≤2	4,278	625 (15.21)	1
	3–4	4,208	735 (17.47)	1.18 (1.05, 1.33)
	≥ 5	731	117 (16.01)	1.07 (0.86, 1.32)
Number of births	1–2	8,037	1,264 (15.73)	0.73 (0.48, 1.12)
	3–4	963	204 (21.18)	1.06 (0.67, 1.66)
	≥ 5	84	9 (10.71)	0.47 (0.21, 1.06)
	None	133	27 (20.30)	1
Number of live births	None	134	27 (20.14)	1
	1–2	8,134	1,275 (15.67)	0.74 (0.48, 1.13)
	3–4	918	199 (21.68)	1.10 (0.70, 1.72)
	≥ 5	31	3 (9.68)	0.43 (0.12, 1.50)
Method of contraception	None	2,261	411 (18.18)	1
	Condom	1,424	178 (12.5)	0.64 (0.53, 0.78)
	Acyeterion	53	9 (16.98)	0.92 (0.45, 1.91)
	IUD	1,975	305 (15.44)	0.82 (0.70, 0.97)
	Sterilization	3,497	599 (17.13)	0.93 (0.81, 1.07)
	Other	28	6 (21.43)	1.23 (0.50, 3.05)
Menopause Status	Yes	27	6 (22.22)	1.47 (0.59, 3.64)
	No	9,190	1,498 (16.30)	1
Disease history	Yes	9	4 (44.44)	4.11 (1.10, 15.33)
	No	9,208	1,500 (16.29)	1
Smoking history	Yes	9,044	1,472 (16.28)	1
	No	220	32 (14.54)	0.97 (0.28, 3.31)
Alcohol consumption history	No	7,440	1,194 (16.05)	1
	Occasionally	1,708	294 (17.21)	1.09 (0.95, 1.25)
	Often	69	16 (23.19)	1.579 (0.90, 2.77)
Sexual partners	≤1	6,024	1,026 (17.0)	1
	≥ 2	68	16 (23.5)	1.49 (1.10,2.65)

*OR, odd ration; HPV infection: including: HPV16, 18, Other HPV types (31,33,35,39,45,51,52,56,58,59,66 and 68) infection; IUD, intrauterine device, Disease history including Gynecological diseases, Vaginal infection, vaginitis*.

### Logistic Regression Analysis of Factors Associated With HrHPV Infection

Seven factors were candidate predictors that were associated with hrHPV infection on univariate analyses. HrHPV infection rates differed significantly according to age [odds ratio (OR) = 1.124, *P* < 0.0001], education level (OR = 1.068, *P* = 0.025), number of births (OR = 0.601, *P* = 0.039), marital status (OR = 1.476, *P* < 0.0001), number of live births (OR = 1.751, *P* = 0.032), age at first delivery (OR = 0.815, *P* = 0.001), and alcohol consumption history (OR = 1.164, *P* = 0.024).

Multivariate analysis confirmed that five variables were independently associated with hrHPV infection, including age, marital status, number of live births, education level, and alcohol consumption history (Table 4; Supplementary Table 1).

**Table 4:** Multivariate logistic regression analysis of factors associated with high-risk HPV infection.

**Variable**	**B Value**	**Wald value**	**Sig value**	**Exp (B)**	**Exp (B) 95% CI**	
					**Lower**	**Upper**
Age (years)	0.117	39.793	0.000	1.124	1.084	1.165
Education level	0.066	5.042	0.025	1.068	1.008	1.131
Age at menarche	0.054	0.893	0.345	1.055	0.944	1.18
Number of pregnancies	0.054	1.234	0.267	1.056	0.959	1.161
Number of births	−0.509	4.268	0.039	0.601	0.371	0.974
Number of live births	0.56	4.582	0.032	1.751	1.048	2.924
Smoking history	−0.18	3.055	0.080	0.836	0.683	1.022
Alcohol consumption history	0.152	5.114	0.024	1.164	1.02	1.328
Disease history	1.259	3.420	0.065	3.523	0.924	13.437
Marital status	0.389	16.998	0.000	1.476	1.226	1.775
Method of contraception	−0.013	0.577	0.448	0.987	0.954	1.021
Age at first pregnancy	0.003	0.005	0.946	1.003	0.916	1.099
Age at first delivery	−0.204	10.733	0.001	0.815	0.722	0.921

### Detection Rates of Cytological Abnormalities in Different Age Groups

The cytological results for the 9,217 women included in the study were as follows: NILM (*n* = 8,020), inconclusive (*n* = 45), ASCUS (*n* = 771, 8.4%), LSIL (*n* = 207, 2.2%), HSIL (*n* = 77, 0.8%), AGC (*n* = 23, 0.2%), ASC-H (*n* = 66, 0.7%), and cervical cancer (*n* = 8, 0.1%). ASCUS or above was not detected in the 21–25 year-old age group, and the detection rate of abnormal cells differed significantly among age groups (*P* < 0.05), with the highest in women aged > 56 years (Table 5).

**Table 5:** Detection of cytological abnormalities according to age group, *N* (%).

**Age (years)**	**NILM**	**ASCUS**	**LSIL**	**HSIL**	**AGC**	**ASC-H**	**CC**	**Inconclusive**	***P*-Value**
21–30	479 (90.9)	35 (6.6)	9 (1.7)	3 (0.6)	0 (0.0)	1 (0.2)	0 (0.0)	0 (0.0)	*P* < 0.0001
31–40	1,960 (87.1)	187 (8.3)	50 (2.2)	17 (0.8)	10 (0.4)	18 (0.8)	0 (0.0)	7 (0.3)	*P* < 0.0001
41–45	1,610 (87.3)	135 (7.3)	54 (2.9)	19 (1.0)	4 (0.2)	11 (0.6)	0 (0.0)	11 (0.6)	*P* < 0.0001
46–50	1,640 (88.2)	145 (7.8)	38 (2.0)	16 (0.9)	1 (0.1)	13 (0.7)	1 (0.1)	6 (0.3)	*P* < 0.0001
51–55	1,340 (85.4)	159 (10.1)	31 (2.0)	12 (0.8)	4 (0.3)	11 (0.7)	3 (0.2)	10 (0.6)	*P* < 0.0001
56–65	991 (84.9)	110 (9.4)	25 (2.1)	10 (0.9)	4 (0.3)	12 (1.0)	4 (0.3)	11 (0.9)	*P* < 0.0001
Total	8,020 (87.0)	771 (8.4)	207 (2.2)	77 (0.8)	23 (0.2)	66 (0.7)	8 (0.1)	45 (0.5)	*P* < 0.0001

*ASCUS, atypical squamous cells of undetermined significance; ASC-H, higher (including low-grade squamous cell-cannot exclude high-grade squamous intraepithelial lesion; LSIL, low-grade intraepithelial lesion; HSIL, high-grade squamous intraepithelial lesion; AGC, atypical glandular cell; NILM, negative for intraepithelial lesion or malignancy; CC, cervical cancer*.

### Detection Rates of Histological Abnormalities in Different Age Groups

Of the 9,217 women who underwent cervical cancer screening, 711 were referred for colposcopy, of whom 250 had abnormal histological findings. We found 108 cases of CIN1 (including 18 cases in Ordos City, 48 cases in Xiangyuan County, and 42 cases in Jinyun County, and Jingning County), 66 cases of CIN2 (including 25 cases in Ordos City, 28 cases in Xiangyuan County, and 15 cases in Jinyun County and Jingning County), 68 cases of CIN3 (including 21 cases in Ordos City, 24 cases in Xiangyuan County, and 23 cases in Jinyun County, and Jingning County), 3 cases of cervical cancer, and 3 cases of vulvar intraepithelial neoplasia among those women with abnormal histological findings. The overall CIN2+ detection rate was 1.45%, of which 0.04%, 0.33%, 0.48%, 0.24%, 0.22%, and 0.17% were in the 21–30, 31–40, 41–45, 46–50, 51–55, and > 56 years age groups, respectively; there was an overall CIN3+ detection rate of 0.77%, with 0.02%, 0.12%, 0.26%, 0.26%, 0.26%, and 0.12% in each age group, respectively. CIN 2/3+ was not detected in women aged 21–25 years, and the highest CIN 2/3+ detection rates were found in the 41–45 years age group, accounting for 32.12% and 17.51% of all cases respectively. The detection rate of abnormal cervical histology differed significantly among the different age groups (*P* < 0.05) (Table 6).

**Table 6:** Rate of detection of histological abnormalities according to age group, *N* (%).

**Age (years)**	**CIN1**	**CIN2**	**CIN3**	**CC**	***P-*value**
21–30	7 (1.3)	2 (0.4)	2 (0.4)	0 (0.0)	0.00
31–40	23 (1.0)	20 (0.9)	11 (0.5)	0 (0.0)	0.00
41–45	13 (0.7)	20 (1.1)	23 (1.2)	1 (0.1)	0.00
46–50	21 (1.1)	11 (0.6)	11 (0.6)	0 (0.0)	0.00
51–55	20 (1.3)	7 (0.4)	13 (0.8)	0 (0.0)	0.00
56–65	24 (2.1)	6 (0.5)	8 (0.7)	2 (0.2)	0.00
Amount	108 (1.2)	66 (0.7)	68 (0.7)	3 (0.0)	0.00

*CIN 1, cervical intraepitelial neoplasia grade 1; CIN 2, cervical intraepitelial neoplasia grade 2; CIN3, cervical intraepitelial neoplasia grade 3; CC, cervical cancer*.

## Discussion

HrHPV infection is closely associated with genital warts and penile, anal, oropharyngeal, and cervical cancers. The elimination of HPV-related cancers, particularly cervical cancer, has attracted global attention as a public health problem (12). The WHO launched the “Global Strategy for accelerating the Elimination of Cervical Cancer as a Public Health Problem” on November 17, 2020, and 194 countries, including China, committed to eliminating cervical cancer for the first time (13). There remains much to achieve to reach the goal of eliminating cervical cancer in China (14). A major problem was low coverage of cervical cancer screening and vaccination with the HPV vaccine; however, some regions, including rural areas, of China, have taken effective measures to prevent and control HPV, such as Ordos City of Inner Mongolia Autonomous Region, Xiangyuan County of Shanxi Province, Jinyun County, and Jingning County of Zhejiang Province. Ordos was the first city in China to implement the national screening plan for women aged 35–64 years and HPV vaccine immunization for girls aged 13–18 years. Based on their experience and study findings, two “National Demonstration Base for Early Diagnosis and Treatment of Cervical Cancer” programs were set up in Xiangyuan County Maternal and Child Health Hospital (rural type) in Shanxi Province in February 2005 (15). Furthermore, Xiangyuan and the counties of Jinyun and Jingning) were the sites for clinical trials of the HPV vaccine and HPV testing kit. Compared with other regions, these areas could be expected to have superior outcomes in terms of prevention and control of cervical cancer, due to the implementation of screening. A 10-year cohort study cervical cancer screening reported reduced rates of HPV infection, particularly infection with HPV16 (16). To assess how the HPV infection rate, the rate of cervical lesion detection, and risk factors influencing the HPV infection rate have changed in these rural areas, we conducted this multicenter, population-based study focused on rural areas, including Ordos, Xiangyuan, Jinyun, and Jingning County, to provide theoretical guidance to further the realization of the plan for the elimination of cervical cancer in rural areas.

In our study, we found that the hrHPV infection rate was 16.3% in the screened population, which was consistent with the findings of Zhao et al. regarding the national screened population but lower than rates reported in other parts of the world, such as Africa (20.9%–23.4%) (17, 18). Combined with our results regarding risk factors, the observed differences may be related to poor health status, early age of marriage, and higher numbers of births, among other factors, associated with hrHPV infection. The HPV 16 infection rate was 3.0%, which was consistent with the findings of the ATHENA study in the USA (2.8%), but it was lower than that reported by the ICO (19, 20). The HPV 18 infection rate was 1.5%, higher than that reported by the ATHENA study (1.0%) (21). The infection rates with HPV 16/18 and the other 12 hrHPV types were 4.3 and 13.6%, respectively. Overall, infection rates with hrHPV, HPV16, 18, 16/18, and the other 12 hrHPV types were consistent with the findings from the national screening population, indicating declining infection rates in these rural areas (Ordos, Xiangyuan, Jinyun, and Jingning County). Except for the infection rates of HPV18, the infection rates of hrHPV, HPV16, 16/18 and other 12 hrHPV types in Inner Mongolia and Shanxi Province were higher than those of in Zhejiang Province, which was indicated that the distribution of hrHPV, HPV16, 16/18 and the other 12 hrHPV types were regional. This may be related to the fact that the women living in pastoral areas of Inner Mongolia or rural areas in Shanxi Province had poor sanitary conditions and premarital sexual behavior (19). In our study, the infection rates of hrHPV, HPV16, 16/18 were higher in Inner Mongolia, Shanxi Province and Zhejiang Province than that in the western region (18) and ICO (20), which may be related to the research subjects coming from the areas with high incidence and mortality of cervical cancer in China. The change in the HPV18 infection rate was not obvious, which may be related to the low infection rate in China. It was suggested that the government should pay attention to the prevention and control of high incidence cervical cancer areas.

Infection rates with hrHPV, HPV16, 18, 16/18, and the other 12 hrHPV types differed significantly according to age (*P* < 0.05), with the peak age of infection at 56–65-years-old in the total mount, Inner Mongolia, Shanxi Province and Zhejiang Province. These findings are inconsistent with the double peak HPV infection rate phenomenon previously reported in rural areas of China (22), which described two peak HPV infection rates in the 25–29 years (14.2%) and 55–59 years (19.3%) age groups. This can likely be attributed to the fact that, in these rural areas (which were clinical trial sites for the HPV vaccine and HPV testing kit), women in the 25–35 years age group underwent HPV vaccine injection, while those aged 35–64 were screened for cervical cancer. Furthermore, the 21–24 years age group was relatively small in our rural population and women tended to marry earlier in rural areas, which may have led to an earlier peak in the HPV infection rate; however, HPV infection in this age group was transient and had no clinical significance. It is established that infection rates exhibit one peak in women aged 21–24 years, which was the highest rate (30.5%) in the ATHENA and ARTISTIC (39.9%) studies (23, 24), subsequently decreasing in women aged ≥ 55 years. Our findings differ from those reports mentioned above; we found that women aged 56–65 years had the highest rate of HPV infection, possibly associated with decreased immunity, a lower natural rate of HPV clearance, and an increased possibility of hrHPV infection in women experiencing menopause or perimenopause (25). Therefore, it is more clinically meaningful to conduct HPV testing for older than younger women, aiming for cervical cancer prevention and treatment in these rural areas (Ordos, Xiangyuan, Jinyun, and Jingning County).

We found that risk factors for hrHPV infection in our population were age, marital status, number of live births, education level, disease history, and alcohol consumption history, consistent with the findings of numerous domestic studies (26, 27). To date, studies conducted in China have unanimously recognized that many sexual partners, more pregnancies, more births, reproductive system diseases, and other factors can increase the risk of infection with hrHPV (28).

In this study, we found that a high number of live births is a risk factor for hrHPV infection, likely due to stimulation and damage of the cervical mucosa, resulting in cervical cell metaplasia or abnormal hyperplasia and changes in estrogen and progesterone levels in the body leading to reduced immunity and increased risk of HPV infection (29, 30). We found that a history of reproductive system infection and gynecological diseases were major risk factors for hrHPV infection, which may be related to the destruction of the cervical mucosal barrier by reproductive system infection, making it easier for HPV to invade and infect the epithelial basal layer, and reducing local immunity in the vagina, causing abnormal differentiation or proliferation of cervical cells (31). Reproductive tract infection with hrHPV is necessary for the development of cervical cancer and CIN; therefore, a key step in cervical cancer prevention is to prevent reproductive tract infections with hrHPV. Menopause was a risk factor for HPV infection in our study, consistent with a report from Smith et al. (32) on postmenopausal women with 7 years of follow-up observation. The proportion of women aged 46–65 years (per-menopause or menopause) in our study was high, and hormone levels in women of this age are relatively disordered, while physiological immunity begins to decline. We found an association between contraceptive methods with HPV infection, which was inconsistent with published literature reports (33). These results suggest that women in our study were insufficiently knowledgeable about contraceptive measures, such as condoms and intrauterine devices, leading to misunderstandings and making it impossible to determine the relationship between contraception and HPV infection rate.

Our data confirm that women with higher education levels had lower hrHPV infection rates, likely because women with higher education levels pay more attention to their health and maintain good personal life and hygiene habits. Previous reports indicate that drinking alcohol is not associated with increased HPV infection rates (34). The women in our study came from areas with large ethnic minority populations. In particular, people with Mongolian ethnicity drink alcohol more frequently than those from other regions. We found that drinking alcohol was a risk factor for hrHPV infection, which warrants further research.

HPV infection rates are higher in married, divorced, and widowed women, which may be related to the number of sexual partners of women in these groups. The HPV infection rate was higher in younger women, consistent with the literature (35). Women aged <20 years may be more likely to be infected with HPV due to immature cervical epithelium repair function and imperfect immune function, as well as early age of initial sexual behavior, which increases the opportunity for HPV infection (36). Due to decreased immune system function and weakened virus clearance ability, the HPV infection rate was higher in older women. Furthermore, women who had ≥ 2 sexual partners had more opportunities to be infected with HPV, consistent with a previous report (37).

We found that the total detection rate of ≥ASCUS was 12.98%, which was higher than those reported by the national “two cancers” screening report (3.93%) (38) and the national special industry project report (5.63%) (39). The detection rates of ASCUS, LSIL, HSIL, AGC, ASC-H, and cervical cancer were 8.4, 2.2, 0.8, 0.2, 0.7, and 0.1%, respectively. Further, the rate of detection of abnormal cytology was high in this study, which may have had several possible causes. First, the cytological diagnoses were all made by doctors with professional training in cytology working at grade A hospitals. Second, the population came from agricultural and pastoral areas, with relatively concentrated ethnic minority populations, or from rural areas that are economically underdeveloped. There were differences rates of abnormal cytology detection among age groups, with the highest rate found in the 56–65 years old group. These data suggest that screening for cervical cancer in older women in rural China should be strengthened. As no abnormal cervical cells were detected in the 21–25 years old group, cervical cancer screening is not advisable for this group. The rate of abnormal cytology detection in the 25–30 years old group was 0.4%, and these women may decide whether to carry out cervical cancer screening according to their economic status.

We found three cases of vulvar intraepithelial neoplasia, which were not analyzed according to age group; however, rates of abnormal cervical tissue differed among age groups. The detection rates of CIN2+ and CIN3+ were 1.45 and 0.77%, respectively, which were lower than those previously published in the literature for rural areas (40, 41). The highest CIN2+ and CIN3+ detection rates were in the 41–45-year-old group, which was similar to the peak age group (40–49 years old). This may be related to differences in geographical regions, methods for cervical cancer screening (liquid-based cytology, HPV detection), tools for screening, population characteristics, age structure, study design, classification used in cytology, and pathology and principles of biopsy sampling, as these factors influence the results of studies of the prevalence of CIN (42). It is clear that the prevalence of cervical lesions is lower in populations with higher rates of screening than in those with lower rates of screening (43). Our study indicates that programs for the prevention and treatment of cervical cancer in these areas are generating initial results. No CIN2+ cases were found in the 21–30 years age group, indicating that cervical cancer screening is not suitable for women in this age group. The detection rates of CIN2+ and CIN3+ were 0.8 and 0.4%, respectively, among women aged 25–30 years, and were highest (32.12 and 17.51%) in women aged 41–50 years. These findings are broadly consistent with the literature (44, 45) and indicate that there should be more focus on cervical cancer screening for middle-aged and older women in rural areas of China.

### Strengths and Limitations

This study has some strengths. The study population was large and a multicenter design was applied, using HPV genotype testing for cervical cancer screening in minority or rural areas of mainland China. To confirm the effects of HPV infection, we chose doctors at local hospitals to perform all screenings, permitting assessment by a superior hospital. Cervical cancer screening has been implemented in rural areas of China for more than ten years; however, the associated risk factors, HPV infection rates, rates of abnormal cervical cytology, and rates of abnormal histology and precancerous lesions in these areas are unclear, and elimination of cervical cancer by screening and prevention programs is expected. Second, our data provide a reference for real-world assessment of the effects of prevention and treatment programs for cervical cancer in rural areas of China.

The study also has some limitations. There was an inevitable loss to follow-up, with a rate of 18.74% in this study; however, analysis of the basic characteristics, main risk factors, and main outcome indicators of the lost population demonstrated that it is unlikely to have influenced our analyses. Furthermore, differences in the cytology, histology, and diagnosis findings of gynecologists in different regions may have had certain impacts on our results.

## Conclusions

The rates of hrHPV and HPV16 infections and cervical lesions in the screened population included in this study were lower than the mean rates in rural China. The infection rates of hrHPV, HPV16, 18, and 16/18 showed a single-peak infection pattern, with a peak infection age of 56–65 years. Risk factors associated with hrHPV infection were: age, history of alcohol consumption, marital status, reproductive diseases, education level, and number of live births. Cervical cancer screening is not recommended for women aged 21–25 years in rural areas, while women aged 26–30 years may decide to undergo screening, according to their economic status. Older women (>30 years old), particularly those aged 41–45 years, are recommended to undergo cervical cancer screening.

## Data Availability Statement

The original contributions presented in the study are included in the article/Supplementary Material, further inquiries can be directed to the corresponding authors.

## Ethics Statement

Central Ethics Approval was obtained from the Cancer Hospital, Chinese Academy of Medical Sciences (No. 16-013/1092). The patients/participants provided their written informed consent to participate in this study.

## Author Contributions

Y-QY, M-YJ, R-MF, MB, WC, and Y-LQ participated in study design, data analysis and visualization, validation of the entire study, and preparation of the manuscript. Y-QY and M-YJ conducted data collection and supervision. Y-QY analyzed the data and write the article. All authors read and approved the final manuscript.

## Funding

The study was funded by Chinese Academy of Medical Sciences (CAMS) Innovation Fund for Medical Sciences (Nos. 2017-I2M-B&R-03 and 2021-I2M-1-004). The authors declare that this study received funding from Roche company. Roche was not involved in the study design, collection, analysis, interpretation of data, the writing of this article or the decision to submit it for publication.

## Conflict of Interest

Y-LQ and WC received grants from the Ministry of Science and Technology of the People's Republic of China during the conduct of the study, and personal fees and non-financial support from Roche. The remaining authors declare that the research was conducted in the absence of any commercial or financial relationships that could be construed as a potential conflict of interest.

## Publisher's Note

All claims expressed in this article are solely those of the authors and do not necessarily represent those of their affiliated organizations, or those of the publisher, the editors and the reviewers. Any product that may be evaluated in this article, or claim that may be made by its manufacturer, is not guaranteed or endorsed by the publisher.

## Footnotes

^1^Available online at: https://m.gmw.cn/baijia/2021-04/21/1302246124.html

## Abbreviations

AGC: atypical glandular cell
ASC-H: low-grade squamous cell-cannot exclude high-grade squamous intraepithelial lesion
ASCUS: atypical squamous cell of undetermined significance
CI: confidence interval
CIN: cervical intraepithelial neoplasia
HPV: Human papillomavirus
hrHPV: high risk HPV
HSIL: high grade squamous intraepithelial lesion
LSIL: low grade squamous intraepithelial lesion
NACCSPRA: National Cervical Cancer Screening Programme in Rural Areas
NILM: negative for intraepithelial lesion or malignancy
OR: odds ratio
WHO: World Health Organization.
