# Supplementary material for: Changes in High-Risk HPV Infection Prevalence and Associated Factors in Selected Rural Areas of China: A Multicenter Population-Based Study
Source: Front Med (Lausanne). 2022 Jul 12;9:911367. doi: 10.3389/fmed.2022.911367 (PMC9319042; doi:10.3389/fmed.2022.911367)
Supplement: Supplementary file 1 [file Table_1.docx]

**Supplemental table 1 Assignment Table of Variables Associated with High-risk HPV Infection**

| Variable | Assignment |
| --- | --- |
| Age (years) | 1 = 21–25; 2 = 26–30; 3 = 31–35; 4 = 36–40; 5 = 41–45; 6 = 46–50; 7 = 51–55; 8 = 56–60; 9 = 61–65 |
| Education level | 1 = Uneducated; 2 = Primary school = junior school = High school = undergraduate and above |
| Age at menarche (years) | 1 = 10–13; 2 = 14–17; 3 = ≥ 18; 4 = Unclear |
| Age at first pregnancy (years) | 0 = No pregnancy; 1 = ≤ 18; 2 = 19–21; 3 = 22–24; 4 = 25–27; 5 = ≥ 28 |
| Age at first delivery (years) | 0 = No deliveries; 1 = ≤ 20; 2 = 21–25; 3 = 26–30; 4 = ≥ 31 |
| Number of pregnancies | 1 = ≤ 2; 2 = 3–4; 3 = ≥ 5 |
| Number of births | 1 = None; 2 = 1–2; 3 = 3–4; 4 = ≥ 5 |
| Number of live births | 1 = None; 2 = 1–2; 3 = 3–4; 4 = ≥ 5 |
| Contraception method | 1 = None; 2 = Condom; 3 = Acyeterion; 4 = IUD; 5 = Sterilization; 6 = Others |
| Disease history | 0 = No; 1 = Yes |
| Smoking history | 1 = Yes; 2 = No |
| Alcohol consumption history | 1 = Never; 2 = Occasionally; 3 = Often |
| Marital status | 1 = Unmarried; 2 = Married; 3 = Widowed; 4 = Separated; 5 = Divorced; 6 = Other |
